# Supplementary material for: Extent of Left Ventricular Mass Regression and Impact of Global Left Ventricular Afterload on Cardiac Events and Mortality after Aortic Valve Replacement
Source: J Clin Med. 2022 Dec 16;11(24):7482. doi: 10.3390/jcm11247482 (PMC9783780; doi:10.3390/jcm11247482)
Supplement: Supplementary file 1 [file jcm-11-07482-s001.zip › jcm-2065669-supplementary.pdf]

Supplemental Table S1. Detailed description of the size and brand for the implanted valves

Variable	Number (Frequency %)
Valve size (n = 95)	
19mm	3(2.5)
21mm	39(32.5)
23mm	38(31.7)
25mm	13(10.8)
27mm	2(1.7)
Valve size for sutureless (n = 25)	
S	8(6.7)
M	9(7.5)
L	5(4.2)
XL	3(2.5)
Brand	
EPIC (porcine)	13(10.8)
Hancock (porcine)	35(29.2)
Mitroflow (bovine)	27(22.5)
Perimount (bovine)	9(7.5)
Magna (bovine)	8(6.7)
Perceval (sutureless)	25(20.8)
St Jude Standard (mechanical)	1(0.8)
St Jude Regeant (mechanical)	2(1.7)
